# Continuous and Autonomous Monitoring of Changes in Left Ventricular dP/dt_max_ Using an Epicardial Accelerometer

**DOI:** 10.1007/s10439-025-03828-6

**Published:** 2025-08-18

**Authors:** Vetle Christoffer Frostelid, Ali Wajdan, Manuel Villegas-Martinez, Lars-Egil R. Hammersboen, Andreas Espinoza, Ole-Johannes H. N. Grymyr, Per Steinar Halvorsen, Ole Jakob Elle, Espen W. Remme

**Affiliations:** 1 The Intervention Centre, Oslo University Hospital, Oslo, Norway; 2 Department of Informatics, University of Oslo, Oslo, Norway; 3 The Faculty of Medicine, University of Oslo, Oslo, Norway; 4 Institute of Surgical Research, Oslo University Hospital, Oslo, Norway

**Keywords:** Accelerometers, Cardiac function, Cardiac mechanics, dP/dt_max_, Contractility, Monitoring

## Abstract

Assessment of the contractile function of the heart typically requires resource demanding techniques, such as invasive left ventricular catheterisation or intermittent medical imaging, and is therefore not readily available for continuous clinical or remote monitoring. Measurement of heart wall motion by use of an epicardially attached three-axis accelerometer has emerged as a promising tool for monitoring cardiac function; however, previous methods have often underutilised the spatial and temporal information contained in the measured signals, potentially limiting its clinical reliability. This work reconstructs the position of an epicardial accelerometer in 3D space in order to enable extraction of indices of cardiac function in a Lagrangian frame of reference. The standard deviation of Lagrangian acceleration throughout a heartbeat, $\sigma _{Acc}$, is introduced as a novel surrogate indicator of contractility as changes in $\sigma _{Acc}$ correlated strongly with changes in the maximum rate of change in left ventricular pressure in animal data (n=29) spanning a variety of haemodynamic conditions. The reported findings of this proof-of-principle study may represent a first step towards long-term monitoring of contractile function and expands on the current repertoire of use for epicardially attached accelerometers as versatile, continuous, and autonomous monitoring devices.

## Introduction

Miniaturised three-axis accelerometers have been incorporated into pacing leads, enabling sensor attachment to the heart wall without additional risk to patient safety and providing a unique opportunity for continuous and autonomous monitoring of cardiac function [1, 2]. Of particular interest is the potential for extracting information on the performance of the left ventricle, the heart chamber responsible for pumping oxygenated blood into the body. Such evaluation may in current clinical practice be performed through invasive left ventricular (LV) catheterisation; however, this is typically carried out in short, dedicated diagnostic or therapeutic procedures and carries with it a low, but not negligible, risk of major complications [3]. Less- or non-invasive alternatives such as medical imaging or arterial/venous pressure analysis are therefore often used, but are typically limited by being intermittent, costly, and/or imprecise [4]. Alternatives for clinical assessment of the contractile function of the left ventricle are especially limited, and novel methods which enable continuous and long-term access to this important aspect of cardiac function may therefore enhance patient management and improve clinical outcomes.

Epicardial accelerometers measure linearly superimposed acceleration at the point of attachment from multiple sources including gravity, macro-scale motion of the heart surface, and smaller vibrations/pressure variations that propagate through the heart and are representative of mechanical phenomena such as valve events, blood flow, and wall oscillations. The end result is a complex but quasi-periodic three-axis waveform that can be hard to visually interpret, with a morphology that may vary considerably due to factors such as anatomy, physiology, sensor position, and sensor orientation [5]. Robust methods for extraction of interpretable indices of function are therefore required, and previous studies have successfully identified a variety of clinically relevant information in the signal. Some have isolated the higher-frequency components of the measured acceleration as the main source of information, likening the epicardial acceleration signals to those obtained from the fields of seismocardiography (SCG) and phonocardiography (PCG), whilst retaining the improved fidelity associated with having the sensor placed directly on the heart [6–8]. Unlike SCG and PCG, epicardially attached three-axis accelerometers also directly measure three-dimensional (3D) cardiac wall motion, which is inherently linked to the heart’s pumping action and has been extensively studied using other measurement modalities. Several studies on cardiac accelerometery have therefore integrated the measured acceleration to velocity and displacement, simplifying the signal into waveforms reminiscent of those seen from echocardiography, thereby improving clinical interpretability and familiarity [9–11]. Attempts have also been made to extract indices from the acceleration signal directly, despite its complex morphology. Two small prospective studies (n=2 and n=4) have found correlations between epicardial acceleration and the maximum rate of change in LV pressure, dP/dt_max_ [12, 13]. dP/dt_max_ is an indicator of contractility [14], a fundamental parameter of cardiac function which accounts for the quality and strength of the LV contraction. A relationship between acceleration and contractility has also been identified using endocardial accelerometers [15, 16] and direct echocardiographic measurements of the myocardium [17–20]. However, the concept has not reached a stage of clinical utility, potentially due to an over-reliance on single-axis measurements and emphasis on peak systolic acceleration as the relevant parameter of contractile function. This work therefore seeks to advance the field through improved utilisation of the physiological and 3D information provided by an epicardial three-axis accelerometer. Presumably, a more contractile heart will have both faster accelerations and decelerations which may not be limited to early systole, but may also be present during ejection, relaxation, and early filling. In this study, we therefore extract the standard deviation of acceleration over a heartbeat, $\sigma _{Acc}$, as a novel index of cardiac function. Furthermore, the index is extracted in a Lagrangian frame of reference, making it independent of sensor implantation orientation and able to utilise the 3D information contained in the signal. As an initial proof of principle, changes in $\sigma _{Acc}$ are investigated for correlation with changes in dP/dt_max_ using animal data. $\sigma _{Acc}$’s ability to capture gradual changes in cardiac function altered through pharmacological manipulation, volume loading, and aortic constriction highlights the potential of the technology as a continuous and autonomous monitoring solution which can provide precise, real-time feedback on cardiac function both in the clinic and remotely.

**Table 1:** A total of 29 animals were included in the study for a total of 22536 heartbeats, or 13561 s, of recorded data across 81 continuous time series

Intervention	Effect on cardiac function	N	Total heart beats	Total time (s)	Mean series duration ± SD (s)
Baseline*	N/A	16	216	165	9.4 ± 0.3
Esmolol*	Suppressive: Reduces the force of contraction by blocking adrenoreceptors	14	2462	1733	124 ± 68
Nitroprusside*	Suppressive: Active vasodilator which leads to a reduction in blood pressure (undesirable for the healthy subjects in this study)	13	2173	1442	111 ± 60
Volume Loading*	Stimulative: Increased blood volume increases preload, which leads to a stronger myocardial contraction through the Frank–Starling mechanism. Excessive loading will eventually be detrimental to function	13	4862	3359	258 ± 190
Epinephrine*	Stimulative: Constricts major blood vessels and increases heart rate and force of contraction	16	5636	2892	181 ± 68
Dobutamine^+^	Stimulative: Increases the force of contraction through stimulation of adrenoreceptors	13	7088	3903	156 ± 75
Aortic Constriction^+^	Rapid constriction of the aorta will in an ideal scenario lead to a rise in systolic left ventricular pressure which is decoupled from changes in dP/dt_max_ for a short period of time before auto-regulatory mechanisms can take effect	4	99	61	15 ± 5

SD: Standard deviation. *: Porcines. ^+^: Canines

## Materials and Methods

The presented framework for monitoring of cardiac function uses a three-axis accelerometer to measure epicardial acceleration and beat-by-beat analysis to quantify cardiac performance through an automated, three-step process: 1) Reconstruction of the 3D displacement that takes place throughout a cardiac cycle. 2) Dimensionality reduction of the 3D displacement into an interpretable, one-dimensional (1D) representation of motion which is independent of the sensor implantation orientation. 3) Extraction of indices of cardiac function.

### Data

This retrospective study uses data from multiple animal studies performed at Oslo University Hospital. All protocols were approved by the institutional and national animal research authorities (Mattilsynet FOTS IDs: 17664, 29818) and carried out in accordance with Norwegian and EU regulations. A total of 29 subjects were included, composed of 16 porcines and 13 canines. All experiments were acute and terminal, and the utilised data were gathered under open-chest conditions and general anaesthesia, with the respirator on. Electrocardiogram (ECG) and LV pressure via invasive micromanometer-tipped catheter were recorded simultaneously to measurements obtained from a three-axis accelerometer sutured to the LV epicardium. Further details of the surgical procedures have been presented previously [21–23]. The end result is a heterogeneous dataset in which multiple sensor models have been used, with data collected by a variety of researchers, and with sensor positioning spanning both the left anterior descending and circumflex artery supplied areas of the LV. The three sensor axes were aligned with the conventional radial, longitudinal, and circumferential directions of cardiac motion in the porcine data, whilst sensor implantation orientation was not verified in the canine data. Table 1 summarises the utilised database and applied interventions. Segments of data were extracted to capture gradual changes in function over time as the interventions were applied, resulting in 81 different time series ranging from 32 s to 835 s long, with dP/dt_max_ ranging from 461 mmHg/s to 11413 mmHg/s. Shorter segments of 10 s to 20 s covering the onset of rapid aortic constriction were extracted in order isolate acute changes in maximum LV pressure (LVP_max_) which were decoupled from changes in dP/dt_max_.

### Data Processing

**Fig. 1:**
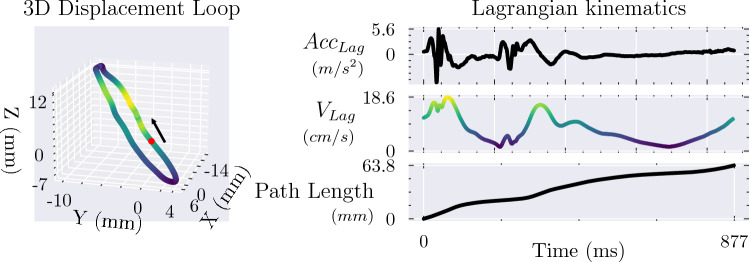
Double integration of the three axes of acceleration allows for determination of the position of the accelerometer in 3D space, which over the course of a cardiac cycle forms a loop due to the contraction and relaxation of the left ventricle. This 3D motion can be reduced to a single dimension through calculation of the Euclidean distance between consecutive points on the loop (Lagrangian kinematics). The colour of the 3D displacement loop corresponds to the calculated Lagrangian velocity, V_Lag_, of the accelerometer. The red dot marks the timing of ECG R-peak in the examined heartbeat and represents the starting point for the kinematic traces.

The recorded time series were segmented based on ECG R-peak detection in order to perform beat-by-beat analysis. By making the assumption that the accelerometer returns to its initial position at the end of the cardiac cycle, the static gravity and drift components of the measured acceleration were removed by subtracting the mean value of the signal for each heartbeat. After the approximate removal of gravity and any potential drift, each acceleration axis was cumulatively integrated to velocity and displacement using the trapezoidal rule. The displacement measured by the three sensor axes thus allowed for the reconstruction of the position of the accelerometer in 3D space, which over the course of a cardiac cycle forms a loop. It should, however, be noted that due to rotation of the accelerometer this method leads to a slight distortion of the reconstructed motion [24, 25]. The Euclidean distance between two points in 3D, $\Vert \mathbf {D_1}-\mathbf {D_0} \Vert = \sqrt{(X_1-X_0)^2+(Y_1-Y_0)^2+(Z_1-Z_0)^2}$, was calculated for consecutive points on the 3D loop, condensing it into a sensor orientation-independent, 1D representation of motion in a Lagrangian frame of reference, i.e. one where the observer follows the accelerometer through space and time. Cumulative summation of the distance between consecutive points results in path length, or the total distance travelled in 3D space, as a function of time. This can be differentiated to yield a velocity magnitude as a function of time which loses any directional information but accounts for all three dimensions of motion, akin to the speed displayed by a car’s speedometer. Differentiation once more yields acceleration (Fig. 1).

This 1D characterisation of the measured 3D acceleration was used to calculate the standard deviation of the acceleration across a heartbeat, $\sigma _{Acc}$, which describes the dispersion of the acceleration around the mean. The acceleration signal contains multiple peaks and troughs, and the standard deviation takes all of these into account to produce a robust measure of the amplitude of the acceleration measured throughout the heartbeat, even though the mean acceleration will be zero as the accelerometer returns to its starting position. Acceleration of a mass implies a net force is acting on it, and the index may therefore be interpreted as a marker of the magnitude of the net force acting on the accelerometer throughout the heartbeat. This will be closely related to the forces generated by the wall itself.

Outlying data points which may have arisen from sources such as incorrect R-peak detection or arrhythmias were smoothed using a Hampel filter with a window size of five samples and threshold of three times the rolling median absolute deviation [26]. Furthermore, a rolling average over five heartbeats was applied to minimise the potential impact of respiration.

**Table 2:** Pearson correlation coefficients (r), limits of agreement (LoA), percentage errors (PE), and standard errors of estimation (SEE) for each intervention. Values are presented as means with 95% confidence intervals, calculated by averaging statistics obtained from individual time series. The mean PE across all interventions was 14% (95% CI [12, 16] and may provide a general estimate of the precision associated with using $\sigma _{Acc}$ obtained from an epicardial accelerometer as a surrogate for dP/dt_max_

Intervention	r	PE (%)	LoA (mmHg/s)	SEE (mmHg/s)
Dobutamine	0.91 [0.87, 0.96]	18 [14, 22]	631 [429, 832]	322 [219, 425]
Epinephrine	0.84 [0.79, 0.89]	22 [17, 26]	636 [336, 936]	325 [172, 478]
Esmolol	0.91 [0.85, 0.96]	12 [8, 15]	124 [86, 161]	63 [44, 82]
Volume Loading	0.66 [0.52, 0.81]	4 [2, 6]	61 [35, 37]	31 [18,44]
Nitroprusside	0.50 [0.24, 0.77]	10 [5, 14]	121 [66, 177]	62 [34, 90]

### Statistical Analysis

Statistical analysis was performed separately for each data series covering the gradual and continuous effect of the interventions in order to eliminate potential inter-subject confounding factors, and more closely mimic a clinical setting in which a patient is continuously monitored over time. Statistical measures were then averaged across all the dose responses per intervention and reported with a 95% confidence interval.

Time series plots showing the concurrent changes in $\sigma _{Acc}$ and dP/dt_max_ allowed for a qualitative assessment of the trending ability of $\sigma _{Acc}$ as a surrogate index of contractility, which was quantified using the Pearson correlation coefficient. Estimates of precision are conventionally obtained using Bland–Altman analysis, allowing for the determination of the 95% limits of agreement. These can be converted to a percentage error (PE) through dividing by the mean measured gold-standard value in order to better represent the measurement error in relation to the range of values covered. $\sigma _{Acc}$ outputs values with units of m/s^2^ and functions as a separate measure of cardiac physiology and not just as a tool to estimate values of dP/dt_max_ in units of mmHg/s. However, calculation of a PE for $\sigma _{Acc}$ in relation to dP/dt_max_ either requires conversion to equal units or normalisation of both parameters. A linear regression between $\sigma _{Acc}$ and dP/dt_max_ was therefore performed for each individual dose response series in order to create a personalised calibration function which converts the measured $\sigma _{Acc}$ to units of dP/dt_max_ and acted as a multi-point calibration of $\sigma _{Acc}$ against the gold standard. This modification to conventional Bland–Altman analysis thus allowed for the assessment of $\sigma _{Acc}$ as a parameter of cardiac performance functionally related to dP/dt_max_. The analysis performed helps quantify the ability of $\sigma _{Acc}$ to *precisely* detect within-patient changes.

The standard error of estimation (SEE) was also calculated in order to enable direct comparison with literature values presented for other alternative indices of contractility. Given a measured value, Y, predicted value, Y’, and N samples: $SEE = \sqrt{\frac{\Sigma (Y-Y')^2}{N}}$ [27].

## Results

**Fig. 2:**
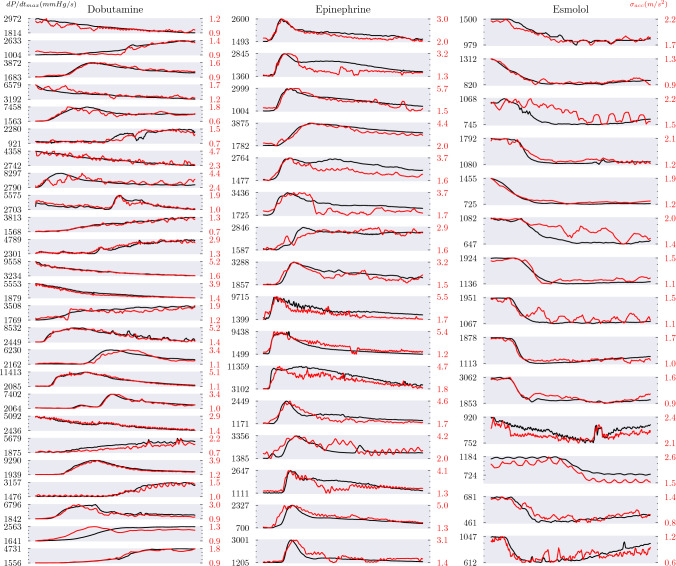
$\sigma _{Acc}$ precisely captured the changes in dP/dt_max_ induced by infusion of dobutamine, epinephrine, and esmolol over time. Black: dP/dt_max_. Red: $\sigma _{Acc}$. Axis labels represent the minimum and maximum values observed in each series. The x-axis represents time elapsed, which varied from series to series, as described in Table 1.

Changes in $\sigma _{Acc}$ closely followed changes in dP/dt_max_ in all dobutamine dose–response series (Fig. 2), leading to a mean Pearson correlation coefficient of 0.91 (95% CI: [0.87, 0.96]). In all cases, the overall trend was captured, with a mean PE of 18% (95% CI: [14, 22]). Similarly, an average correlation coefficient of 0.84 (95% CI: [0.79, 0.89]) and PE of 22% (95% CI: [17, 26]) was found across the 16 epinephrine responses (Table 2). Dobutamine and epinephrine both lead to a substantial increase in dP/dt_max_ through stimulation of adrenoreceptors which increase the force of contraction, although epinephrine comparatively has a greater positive chronotropic effect, as well as greater but dose-dependent vasoconstrictive and dilating effects [28, 29]. $\sigma _{Acc}$ therefore appears to be a robust surrogate for monitoring changes in dP/dt_max_ at elevated contractility, and the correlation remains strong even when heart rate and afterload are also altered.

**Fig. 3:**
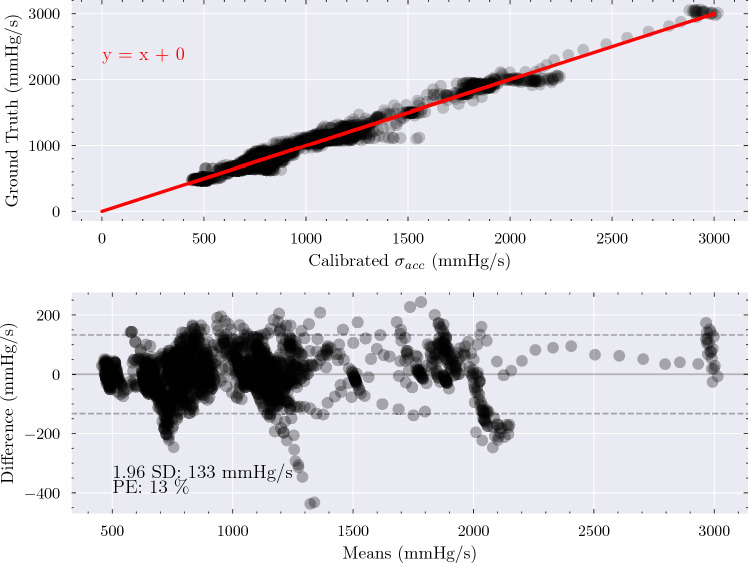
Top: Comparison of dP/dt_max_ measured invasively and dP/dt_max_ obtained from $\sigma _{Acc}$ after the application of a personalised calibration function for all esmolol dose responses revealed a strong functional relationship between the two. Bottom: Bland–Altman analysis showed consistently distributed differences, with limits of agreement of 133 mmHg/s corresponding to a percentage error of 13%, suggesting $\sigma _{Acc}$ can be used to precisely monitor patients with weak contractile function.

Changes in $\sigma _{Acc}$ also trended closely with changes in dP/dt_max_ in the responses to esmolol infusion, resulting in an average correlation coefficient of 0.91 (95% CI: [0.85, 0.96]) and PE of 12% (95% CI: [8, 15]), corresponding to limits of agreement of 124 mmHg/s. $\sigma _{Acc}$ therefore also appears to hold potential as a surrogate for monitoring changes in dP/dt_max_ in patients with weak contractile function. Figure 3 compares the dP/dt_max_ values obtained from a personalised calibration of $\sigma _{Acc}$ with dP/dt_max_ values measured invasively for all the esmolol response series. There is a strong functional relationship between the two, suggesting that the use of linear, personalised calibration functions was appropriate in the range of cardiac function examined and that $\sigma _{Acc}$ has the ability to precisely monitor within-patient changes in cardiac function.

**Fig. 4:**
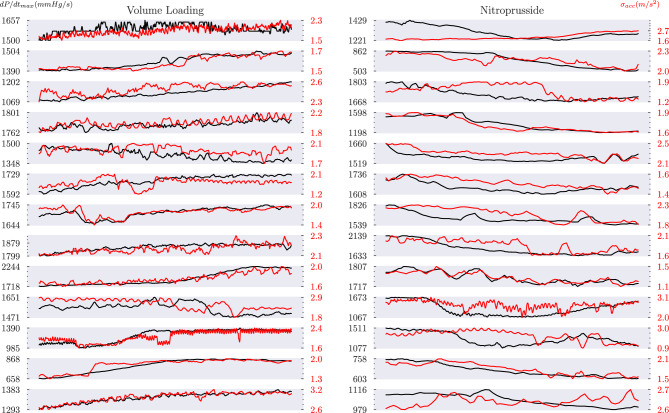
Volume loading and nitroprusside infusion induced a comparatively small change in dP/dt_max_, often of similar magnitude to the observed limits of agreement. The correlation between $\sigma _{Acc}$ and dP/dt_max_ is therefore obfuscated by the limits of the precision of $\sigma _{Acc}$, although in most cases the general trend is still captured. Black: dP/dt_max_. Red: $\sigma _{Acc}$. Axis labels represent the minimum and maximum values observed in each series. The x-axis represents time elapsed, which varied from series to series, as described in Table 1.

Compared to the above interventions, nitroprusside infusion and volume loading induced minor changes in dP/dt_max_ (Fig. 4). The PEs remained low, at 4% and 10% for volume loading and nitroprusside, respectively. However, the corresponding limits of agreement were often of similar magnitude to the observed changes in dP/dt_max_, leading to obfuscation of the correlation analysis and weaker correlation coefficients of 0.66 (95% CI: [0.52, 0.81]) and 0.50 (95% CI: [0.24, 0.77]) for volume loading and nitroprusside, respectively. Nonetheless, over the course of the measurements an overall reduction or increase in dP/dt_max_ was captured by $\sigma _{Acc}$ in 23 out of 26 cases.

**Fig. 5:**
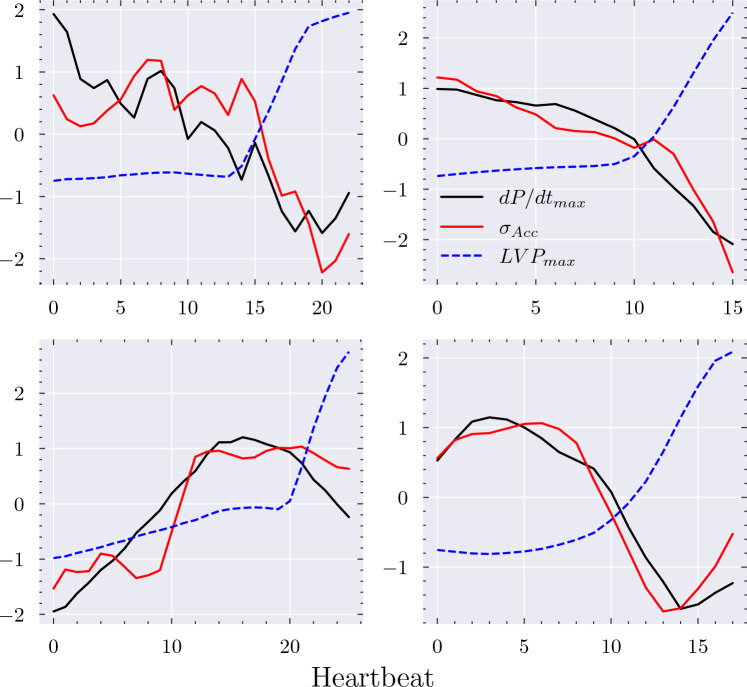
Constriction of the aorta leads to a rapid rise in maximum left ventricular pressure, LVP_max_. In the four examined subjects, $\sigma _{Acc}$ continued to closely follow dP/dt_max_, highlighting its ability to differentiate changes in the heart’s contractile ability from changes in LVP_max_; two factors which are otherwise usually strongly correlated. All time series were z-normalised to fit on the same scale.

An increase in dP/dt_max_ is typically highly correlated with an increase in LVP_max_, as a stronger contraction will generate a higher pressure in otherwise similar haemodynamic conditions. Rapid aortic constriction effectively leads to an increased afterload and a stronger force opposing the opening of the aortic valve, preventing effective ejection of blood and leading to the generation of a higher pressure within the ventricle prior to aortic valve opening. Various auto-regulatory mechanisms will quickly activate so that cardiac function adjusts to this sudden change in haemodynamics; e.g. reduced ejection may lead to increased preload which increases the force of contraction and improves the ejection. However, in the short term the increased afterload will lead to a higher LVP_max_ without a corresponding increase in dP/dt_max_. In the 4 subjects in which aortic constriction was applied in this study, LVP_max_ increased by an average of 35 mmHg or 36%, whilst dP/dt_max_ decreased by an average of 60 mmHg/s or 4%. Changes in $\sigma _{Acc}$ trended closely with changes in dP/dt_max_ and did not follow the rapid increase in LVP_max_ which occurred upon aortic constriction (Fig. 5). $\sigma _{Acc}$ therefore appears to be related to *how* the pressure in the ventricle is generated and not directly to the absolute pressure itself.

## Discussion

**Fig. 6:**
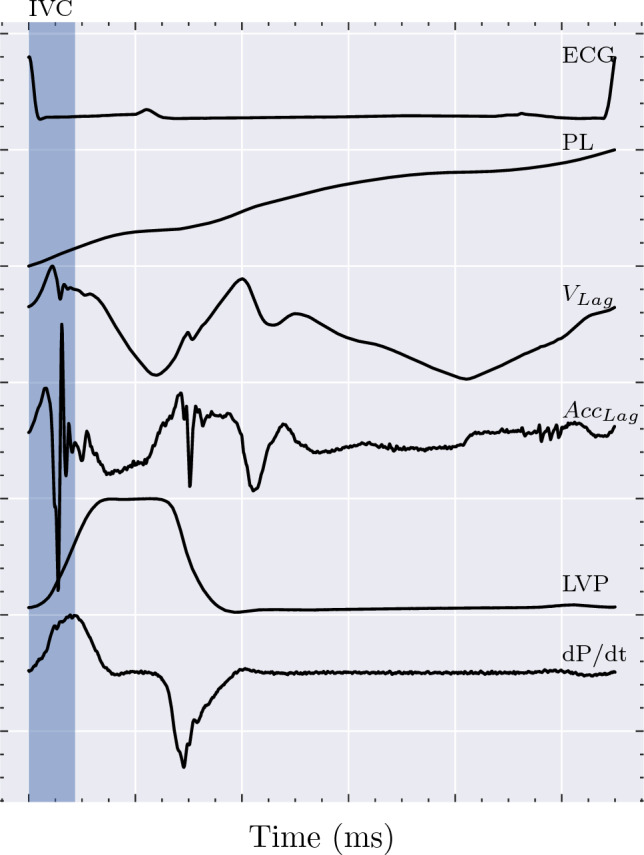
A substantial portion of pressure generation in the left ventricle takes place during the isovolumic phase of contraction (IVC). Despite the constant volume of the cavity, motion of the epicardium is observed by the accelerometer directly after electrical activation due to myocardial shortening, and epicardial acceleration can therefore be used to gain insights into this phase of pressure generation. IVC has been estimated as the duration of R-peak until dP/dt_max_ to illustrate the approximate duration of this phase of the cardiac cycle. PL: Path length, i.e. total distance travelled in 3D space over time. V_Lag_: Lagrangian velocity; derivative of PL. Acc_Lag_: Lagrangian acceleration; derivative of V_Lag_.

This proof-of-principle animal study demonstrates the feasibility of extracting a beat-to-beat index from an epicardially attached three-axis accelerometer which can be used to continuously and autonomously monitor cardiac function. Correlation of accelerometer indices to existing clinical indices may enable an improved understanding of their physiological interpretation and lower the threshold for clinical usage of the technology. This work therefore presents changes in $\sigma _{Acc}$ as a potential surrogate indicator of changes in contractility, supported by a correlation to changes in dP/dt_max_ in animal data. Contractility is a fundamental parameter of cardiac function which is not readily available in current clinical practice [30]. Whilst dP/dt_max_ is widely accepted an index of contractility, it should be noted that it is preload dependent. End-systolic elastance (E_es_) is generally considered a better and less load-dependent index of contractility; however, its measurement requires transient preload reduction and complex pressure–volume loop analysis and is intermittent. Using E_es_ as the gold standard was therefore not feasible using the current material, but should be explored in future research.

The relationship between $\sigma _{Acc}$ and contractility, or the myocardium’s inherent ability to generate a force of contraction, may be understood through Newton’s second law, ${\textbf {F}}=m{\textbf {a}}$, as a stronger net force is proportional to a stronger acceleration. A substantial amount of the pressure generation in the left ventricle takes place during the isovolumic phase of the contraction, i.e. when the mitral and aortic valves are both closed, with dP/dt_max_ typically occurring near the time of aortic valve opening [31–33]. However, despite the isovolumic state of the LV cavity, tissue Doppler echocardiography has revealed that there is a substantial amount of motion related to mitral valve closure and the pressure-generating contraction of the myocardium, and this was also observed by the epicardial accelerometers used in this work (Fig. 6) [34]. Epicardial accelerometers are therefore able to extract physiologically relevant information pertaining to this phase of pressure generation. Furthermore, any motion observed after the aortic valve opens and the LV volume decreases will also be linked to the force of contraction, although potentially affected by afterload to a greater extent. Faster speed of contraction has also been shown to be strongly correlated to faster relaxation of the myocardium such that increased contractility is effectively linked to reduced resistance to filling [35]. Additionally, greater systolic compression leads to greater restoring forces, and both these factors combined therefore lead to increased speed of filling and wall motion (e’) [36]. Improved contractility may therefore also affect the acceleration observed during ventricular filling post mitral valve opening. By using the standard deviation of the Lagrangian representation of the measured acceleration across the whole cardiac cycle as an index, information from all three spatial dimensions and the temporal dimension can be condensed into a single, robust value which is able to represent contractility by retaining a substantial amount of the relevant physiological information contained in the measured signal.

Whilst correlated, the mechanics which underlie changes in $\sigma _{Acc}$ and changes in dP/dt_max_ are different. $\sigma _{Acc}$ is a measurement of the motion of the epicardium, i.e. cardiac kinematics, whilst dP/dt_max_ is a measurement of the pressure generation inside the cavity. Some discrepancy between the two may therefore be expected. In particular, the abrupt drop in afterload associated with vasodilation induced by nitroprusside infusion appeared to lead to a rapid drop in dP/dt_max_, but had a lesser or even contradictory short-term effect on the acceleration of the epicardium. This may be related to the reduced force opposing the contraction of the ventricle and aortic valve opening. However, after a short period of time in which various auto-regulatory mechanisms may have activated, dP/dt_max_ and $\sigma _{Acc}$ typically converged to a reduced value. Interpretation of $\sigma _{Acc}$ should therefore be performed whilst bearing in mind that it is a separate and novel physiological measurement of cardiac function. The response series resulting from volume loading suggest that $\sigma _{Acc}$, like dP/dt_max_, is preload dependent. Normalisation of load-dependent indices of contractility to preload has been shown to improve agreement with E_es_ [37]. Features derived from heart sounds measured by epicardial accelerometers have previously been correlated to preload [6]. This may be used for preload correction of $\sigma _{Acc}$, potentially enabling an improved, continuous single beat index of contractility.

The clinical application of $\sigma _{Acc}$ requires further investigation. $\sigma _{Acc}$ may be used as a stand-alone index of cardiac function or used in combination with a transfer function to estimate an existing clinical measure such as dP/dt_max_. This study evaluated $\sigma _{Acc}$ with emphasis on quantifying its ability to continuously monitor cardiac function with a high degree of precision. For that purpose, a personalised calibration function was derived based on invasive measurement of dP/dt_max_. This may be replicated in clinical practice if LV catheterisation is performed during the implantation of the sensor, but represents an idealised scenario which may not always be feasible. The correlation analysis revealed a linear relationship between changes in $\sigma _{Acc}$ and dP/dt_max_. A patient-specific calibration function may therefore also be generated using a one- or two-point calibration against an existing, intermittent index of contractility. Such patient-specific calibration is often used in current clinical practice; e.g. pulse contour analysis of the arterial pressure waveform is calibrated against thermodilution to enable continuous cardiac output monitoring. Alternatively, future work may seek to define reference values for $\sigma _{Acc}$ as a direct index of cardiac function or generate a population spanning transfer function which converts $\sigma _{Acc}$ to dP/dt_max_. Both these scenarios would benefit from further investigations into the effect of sensor positioning, a factor which may affect both the direction and magnitude of the measured motion. The Lagrangian approach utilised for extraction of $\sigma _{Acc}$ is invariant to the implantation orientation of the sensor and utilises information from all three axes of motion. It may therefore minimise inter-subject variation caused by regional differences in motion direction. Furthermore, recipients of cardiac implants in which an accelerometer could be incorporated will likely also receive some form of imaging based analysis of cardiac function. Intermittent 4D imaging of the ventricle can potentially be used to calculate $\sigma _{Acc}$ for multiple points of the cardiac wall, allowing for the determination of a geometry wide mean value. This could be used to scale the continuous single point measurement obtained by an accelerometer and reduce the inter-subject variation caused by regional differences in motion magnitude. In this retrospective study, measurements obtained from sensors placed in both the circumflex and left anterior descending artery supplied areas of the LV were successfully used to monitor changes in cardiac function. This suggests accelerometer positioning is not critical for monitoring of within-patient changes; however, this study is limited by lack of dedicated investigation into sensor positioning. Further investigation into this aspect of cardiac accelerometry is therefore needed, but falls outside of the scope of this work. When monitoring cardiac function it is often crucial to know whether a clinical intervention is leading towards one direction or another [38]. The method’s ability to precisely monitor within-patient changes independently of sensor positioning or calibration is therefore valuable, and may be utilised in clinical practice. Based on the presented results, a drop in $\sigma _{Acc}$, regardless of the starting value, would provide a strong indication that dP/dt_max_ has also dropped, and may be cause for a more thorough evaluation of cardiac function and treatment parameters in the monitored patient.

Echocardiographic measurement of mitral valve regurgitation can be used to estimate dP/dt_mean_, with associated clinical classifications of normal (>1200 mmHg/s), borderline (800-1200 mmHg/s), reduced (500-800 mmHg/s), and severely reduced (< 500 mmHg/s) contractile function. This method has a reported SEE of 50-90 mmHg/s [39, 40]. Comparatively, the average SEE found across the 14 examined esmolol dose responses was 63 mmHg/s (95% CI: [44, 82]). When monitoring within-subject changes in function, $\sigma _{Acc}$ may therefore be able to detect a drop in function with the precision required to differentiate the clinical classification intervals of 300 to 400 mmHg/s. Furthermore, it would do so continuously and autonomously, without requiring substantial mitral valve regurgitation or manual measurements and analysis post implantation. This may in the future enable continuous evaluation of drug or fluid response, or facilitate improved, long-term assessment of cardiac contractility and autonomous monitoring systems which can alert healthcare practitioners to a reduction in function. An accelerometer incorporated into a permanent pacing lead which can capture changes in $\sigma _{Acc}$ over days, weeks, or months may allow clinicians to amend treatment plans, minimise home visits, or intervene when needed without relying on planned follow-ups at a hospital.

The mean PE across all dose responses and interventions was 14% (95% CI [12, 16]) and may serve as a general measure of the precision of the presented method. Importantly, the PE remained small for low values of dP/dt_max_, which is essential for differentiation of function in patients with weak contractile function. Low, outlying PE values (minimum 0.6%) showcase the potential of the method for precise monitoring of changes in dP/dt_max_. The range in precision and high, outlying values (maximum 51%) may plausibly be related to practical circumstances such as how well the accelerometer was attached to the surface of the heart, tension in the cable connected to the accelerometer, or interaction between the sensor and the surrounding pericardium or chest. These factors may lead to distortion of the measured motion, and must therefore be carefully managed in a clinical setting and through hardware design in order to optimise monitoring of cardiac function using epicardial accelerometers. Incorporation of motion sensors into existing cardiac implants such as temporary pacing leads, or the LV leads of cardiac resynchronisation therapy devices, may realistically enable measurement of LV motion in clinical practice with minimal additional risk. This may be a relevant first step to determine if stand-alone accelerometer implants can be warranted in the future.

Generally, the peak amplitude of the measured acceleration has been extracted as the relevant index for analysis when myocardial acceleration and contractility have been correlated in previous research, regardless of modality and measurement location. The standard deviation of acceleration across the whole cardiac cycle used in this study was observed to perform better as an index than the maximum value for all interventions (e.g. r = 0.84, 95% CI: [0.79 0.89] vs 0.59, 95% CI: [0.43 0.75] for the epinephrine data), which may be attributed to it taking into account more relevant physiological information as described above, as well as a greater resistance to noise and variance in the signal. An analysis of the inter-beat variance of $\sigma _{Acc}$ in baseline conditions revealed that the mean inter-beat standard deviation for $\sigma _{Acc}$ across all subjects was 7% (95% CI: [6, 9]) relative to the mean value. This contrasted max epicardial acceleration as an index which had a mean inter-beat standard deviation of 14% (95% CI: [11, 16]). There is therefore a tangible benefit of increased precision associated with using the acceleration across the entire heartbeat as a functional index, although future work may aim to explore whether isolating physiologically relevant segments of the heartbeat for acceleration analysis may be beneficial. Comparatively, dP/dt_max_ was more stable at 2% (95% CI: [2,3]). The higher inter-beat variance of $\sigma _{Acc}$ may possibly be partially explained by respiratory motion leading to failure of the assumption that the accelerometer returns to its starting position at the end of a cardiac cycle. A moving average over five beats was used to minimise this effect in the analysis presented, but oscillatory trends which appear related to respiratory motion are still apparent in many cases. Combining the accelerometer with a gyroscope and magnetometer to form a complete inertial measurement unit may improve the motion reconstruction and potentially minimise artefacts related to respiratory motion.

This work encourages further studies which may help translate the technology towards clinical usage and long term, autonomous home monitoring of cardiac function. Further evaluation of the clinical application and potential benefits of the technology is needed and may aim to improve on the limitations of this work. In addition to the limitations discussed above, this study was conducted using data acquired under open-chest conditions and general anaesthesia. This may not accurately represent the typical clinical scenario for patients. A previous study performed with closed-chest pigs showed that chest-closure may affect the measured motion pattern, particularly in the apical region [22]. However, changes in cardiac function still induced changes relative to the baseline motion patterns, allowing for monitoring of cardiac function. Due to using open-chest data, variations in sensor positioning, and the fact that data from two animal species were used, reference values for $\sigma _{Acc}$ or a population wide transfer function which converts $\sigma _{Acc}$ to dP/dt_max_ have not been presented, and these factors represent limitations of the study. Epicardial accelerometer measurements are also highly sensitive to patient movement. Currently, monitoring of function using epicardial accelerometers would therefore be limited to when the patient is at rest. Future work may explore algorithms for correction of patient movement using a secondary sensor placed externally on the chest or embedded in an implanted pacing control device.

$\sigma _{Acc}$ has been presented as a stand-alone indicator of cardiac function; however, a variety of functional accelerometer indices have been extracted in previous work and represent different aspects of the underlying physiology of the heart. Algorithms which combine the information from multiple indices may therefore further improve the accuracy of a functional assessment performed using epicardial accelerometers, as is done in current clinical practice when other modalities are used, e.g. for echocardiographic assessment of diastolic LV function [41]. Combined with previous studies that have used epicardial accelerometers to detect ischaemia and valve events and extract indices correlated with preload, LV stroke work, and myocardial strain [5, 9, 42], this work advances the current status of epicardial accelerometers as versatile tools for continuous monitoring of cardiac function. Epicardial accelerometers may in the future consequently be able to effectively supplement current clinical methods and provide medical practitioners with additional information which may be used to aid clinical decision-making.
